# Carbon dots alleviate salinity stress and promote growth in tomato (*Solanum lycopersicum*)

**DOI:** 10.3389/fpls.2026.1772618

**Published:** 2026-03-18

**Authors:** Sharareh Rismani, Mansour Shariati, Didier Reinhardt, Seyed Morteza Javadi Rad

**Affiliations:** 1 Department of Plant and Animal Biology, Faculty of Biological Sciences and Technologies, University of Isfahan, Isfahan, Iran; 2 Department of Biology, University of Fribourg, Fribourg, Switzerland; 3 Department of Cell and Molecular Biology and Microbiology, Faculty of Biological Sciences and Technologies, University of Isfahan, Isfahan, Iran

**Keywords:** carbon dots, chlorophyll *a* fluorescence, NaCl, salt stress, *Solanum lycopersicum*, tomato

## Abstract

**Introduction:**

Soil salinity represents an environmental stress that substantially lowers plant performance, thereby reducing crop yield and threatening food security in many arid environments.

**Methods:**

Here, we describe the response of tomato (*Solanum lycopersicum*) to high levels of NaCl, and we test the potential of carbon dots (CDs) to alleviate salt stress. Hydrothermally synthesized CDs were characterized by electron microscopy and Fourier transform infrared (FTIR) spectroscopy to identify chemical groups such as hydroxyl, carboxyl, and amine groups. Zeta potential analysis revealed a negative surface charge.

**Results:**

While salt stress compromised various physiological parameters in tomato plants, CD treatment of salt-stressed plants enhanced salt tolerance as indicated by increased dry weight, chlorophyll content and relative water content. CDs also decreased energy dissipation and increased PSII fluorescence indices such as the quantum yield of electron transport, and the performance index in salt-stressed plants. Furthermore, CDs enhanced the content of soluble carbohydrates and contributed to restore the K^+^/Na^+^ balance in plants under salt stress. Salt-responsive genes (*SOS1*, *SOS2*, *SOS3*, *NHX4*, and *NHX2*) were induced to a lesser extent when salt-stressed plants were treated with CDs, consistent with the observation that CDs protected plants against salt stress.

**Discussion:**

Taken together, these findings show that CDs can mitigate salt stress in tomato.

## Introduction

One of the most important problems associated with the growing global population is ensuring sufficient food supply. Given the critical role of agriculture in feeding the human population, it is essential to address the challenges of this sector, particularly in view of the increasing environmental stresses associated with global warming. Central threats to food security are water deficit and soil salinity (Jia et al., 2013), which represent major constraints to crop yield and quality in many regions worldwide (Sánchez-Barrena et al., 2005). Modern agricultural research aims to develop simple, affordable, and environmentally safe strategies to counteract the negative impacts of salt stress on crop production. Increasingly, nanobiotechnology is contributing to solutions for sustainable agriculture. Carbon dots (CDs) are nanoparticles ranging from 1–10 nm, which offer various advantageous features. These characteristics include high water solubility, biodegradability, the ability to access plant tissues, and strong fluorescence, allowing them to be traced in biological systems (Hoang et al., 2019; Liu et al., 2019; Li et al., 2010). CDs have the potential to increase stress resistance, particularly salt tolerance in plants (Sarkar et al., 2022; Li et al., 2020a).

Soil salinity increases osmotic stress in plants and causes stomatal closure, resulting in decreased CO_2_ uptake, photosynthesis, and growth. In addition, the accumulation of Na^+^ and Cl^−^ ions in plant cells has direct effects by disrupting ion homeostasis (Guo et al., 2022). This impairs enzyme function, water uptake, and chlorophyll biosynthesis, ultimately disrupting the structure and function of photosystem II (Baker, 2008; Strasser et al., 2004).

Plants have evolved several defense mechanisms to counteract the detrimental impacts of salt stress. The activation of the Salt Overly Sensitive (SOS) signaling pathway, mediated by SOS1, SOS2, and SOS3 (Gupta et al., 2021; Jia et al., 2013) represents a central protective mechanism against high salt conditions. Activation of this pathway results in increased cytoplasmic Ca^2+^ which in turn activates SOS3. SOS3 induces the activity of the SOS2 kinase which then activates the Na^+^/H^+^ antiporter SOS1 in the plasmalemma. In addition, the sodium-hydrogen exchanger (NHX) proteins NHX4 and NHX2 are activated (Guo et al., 2022; Belver et al., 2012; Huertas et al., 2012). Na^+^/H^+^ antiporter activity of SOS1, NHX4 and NHX2 mediates the extrusion of Na^+^ from the cytosol or compartmentalization in the vacuole. This ultimately contributes to counterbalancing the disturbance of K^+^/Na^+^ homeostasis in plant cells (Jia et al., 2013). Besides endogenous protective mechanisms, recent findings suggest that CDs can also contribute to crop protection against salt stress (Sarkar et al., 2022; Li et al., 2020a). Based on these findings, the present study explores the potential of CDs to mitigate salt stress in tomato at the physiological and transcriptional levels.

## Materials and methods

### Synthesis of CDs

To produce nitrogen-doped CDs by the hydrothermal method, the procedure described by Arab et al. (2020) was employed. To perform chemical reactions under high temperature and pressure, a custom autoclave was constructed from a steel cylinder and a heat-resistant polytetrafluoroethylene (PTFE) Teflon chamber with a volume of 25 mL. Synthesis proceeded with a mixture of 1 g citric acid in 850 μL ethylenediamine and 10 mL of distilled water at 180°C for 5 h. After cooling, the mixture was centrifuged at 15,700 g (58010/5810 R, Eppendorf, Germany) for 10 min. The supernatant was immediately filtered through a 0.22 μm cellulose acetate syringe filter and then purified by dialysis (Biotech CE Dialysis Tubing 500D, 31 mm, 10 m) for 8 h. To disperse CD particles and avoid aggregation, the CD suspension was ultrasonicated (Ultrasonic Cleaner Company, model vCLEAN1-L2, Iran) for 20 min and then immediately lyophilized. To prevent CDs from undergoing physicochemical reactions with the environment before plant experiments, the resulting powder was stored in a vacuum-sealed glass container in a dark place at -4°C.

### CD characterization

The maximum excitation and emission wavelengths of CDs were determined by absorption and fluorescence spectra using an FP-6200 spectrofluorometer (Jasco, Japan). To visualize fluorescence, an image of CDs suspended in water was taken with a still camera under UV and visible light. The size and morphological features of the CDs were characterized by transmission electron microscopy. Briefly, an aqueous suspension of CDs was sonicated (S3000, Misonix, USA) for 15 min to prevent aggregation of individual particles. Then, a drop of the suspension was transferred to a formvar carbon film on a copper grid (300 mesh; EMS, USA) and dried thoroughly at room temperature. The sample was analyzed with an EM10C transmission electron microscope (Zeiss, Germany) at an acceleration voltage of 100 kV. X-ray diffraction (XRD) patterns were obtained using a D8 Advance Diffractometer (Bruker AXS, Germany), while the surface charge of the CDs was measured with a SZ-100 zeta potential analyzer (Horiba, Japan). To characterize surface functional groups of CD structures, Fourier transform infrared spectroscopy (FTIR) was performed (FT/IR-6300, Jasco, Japan).

### Plant cultivation and treatment

Tomato seeds were surface-sterilized with 70% ethanol for 1 min followed by treatment with 1% sodium hypochlorite for 20 min. Seeds were sown in small peat moss–containing pots and kept in the dark and humid conditions for 72 h. Germinated seeds were maintained in a growth room at 25 ± 2°C with a 16/8 h light/dark cycle under an illumination intensity of 100 µmol photons.m^−^² s^−^¹ (PAR Quantum Sensor, Hansatech, UK). They were irrigated twice a week. After two weeks, seedlings were transplanted into hydroponic culture under the conditions mentioned above. Micro- and macro-elements were administered according to Arnon and Johnson (1942). The medium was adjusted to pH=6 and aerated using air pumps. After one week of adaptation to the hydroponic conditions, seedlings of comparable size were selected and transferred to hydroponic nutrient solutions containing CDs, NaCl, and combinations of CDs and NaCl for one week. To keep CD particles dispersed, the suspension was ultrasonicated after CDs synthesis and purification, and before treatments. During experiments, plants were removed twice daily from the nutrient solutions containing CDs for few minutes and the solutions were mixed well using magnetic stirrers. CD suspensions were stable over many months, as no precipitation was observed and their fluorescence characteristics remained constant. All treatments were performed in four independent biological replicates. To measure physiological and molecular parameters, leaf and root samples were harvested at the end of the experiment. To determine appropriate experimental conditions, different concentration ranges of CDs (0, 30, 60, 120, and 240 mg·L^−^¹) and NaCl (0, 50, 100, and 150 mM) were tested, and based on the effects on seedling dry weight, 30 mg·L^−^¹ CDs and 100 or 150 mM NaCl were chosen for further experiments.

### CD transport to the shoot

To assess uptake and transport of CDs in tomato plants, the intense fluorescence of CDs was traced in tissues. Plants were treated via the root system by hydroponic application with 30 mg·L^−^¹ CDs for 1 week. Leaflets of control and CD-treated plants were imaged under identical conditions under visible and UV light with a smartphone.

### Dry weight measurement

Intact plants including roots and shoots of treated and untreated plants were harvested and dried in an oven at 70°C. After 72 h, the dry weight (DW) was measured.

### Relative water content measurement

The fresh weight (FW) of one leaf per plant excised from the same position at the leaf base was measured. Then, the samples were soaked in distilled water for 24 h, and the turgid weight (TW) was measured. Subsequently, tissues were dried at 60°C for 72 h and weighed as dry weight (DW). Relative water content (RWC) was calculated by the following formula (Weatherley, 1950): RWC (%) = (FW - DW)/(TW - DW) × 100.

### Chlorophyll assay

Chlorophyll content was determined by the method described by Wellburn (1994). Briefly, 100 mg of fresh leaves were homogenized in 10 mL of 80% acetone under dim light. The mixture was centrifuged at 9660 g for 10 min at 4°C (58010/5810, Eppendorf, Germany). The absorbance of the supernatant was measured at 663, 646, and 470 nm using a multimode microplate reader (Agilent Biotek Synergy HTX, USA). Total chlorophyll content was calculated in terms of µg·mg^−^¹ FW using the following equations:


$$\text{Chl a }(\text{µg}\cdot {\text{mg}}^{-1})=12.21\;(\text{A}663)–2.81\;(\text{A}646)$$

$$\text{Chl b }(\text{µg}\cdot {\text{mg}}^{-1})=20.13\;(\text{A}646)–5.03\;(\text{A}663)$$

$$\text{Total Chl }(\text{µg}\cdot {\text{mg}}^{-1})=\text{Chl a}+\text{Chl b}$$

### Carbohydrate analysis

Ten milligram of dried leaf tissue was homogenized with 10 mL of warm distilled water and filtered through filter paper. The filtrate was used for measurement of soluble carbohydrate with the phenol–sulfuric acid method (Dubois et al., 1956). Briefly, 0.5 mL of filtrate was mixed with 0.5 mL of phenol and 2.5 mL of 96% sulfuric acid, vortexed, and incubated at room temperature. After 30 min, the absorbance of the solution was measured at 490 nm using a multimode microplate reader. Soluble carbohydrate content was calculated as µmol**·**g^−^¹ FW using a glucose standard curve ranging from 0.125 to 10 mM.

### Na^+^ and K^+^ quantification

A wet-ashing method based on triacid digestion was used to determine the Na^+^ and K^+^ content (Sahrawat et al., 2002). Five hundred milligrams of dry root powder was transferred to 125-mL conical flasks. Then, 12 mL of a triacid mixture composed of nitric acid, sulfuric acid, and perchloric acid (9:2:1, v/v) was added. The mixtures were heated on a hot plate for 2–3 h until tissues were completely dissolved, and the solutions became colorless. After cooling, the sample volumes were adjusted to 125 mL using distilled water and filtered through Whatman filter paper. The resulting solutions were used to determine Na^+^ and K^+^ concentrations using a flame photometer (Corning Flame Photometer 410, UK). Serial dilutions of NaCl and KCl solutions (0.1–1 mM) were prepared as standard curves for Na^+^ and K^+^ to determine absolute ion concentrations (mmol·g^−^¹ DW).

### Photosynthetic parameters

The Plant Efficiency Analyzer (Handy PEA fluorimeter, Hansatech Instruments Ltd, UK) was employed to measure the rapid chlorophyll *a* fluorescence transient with a high resolution of 10 µs (118 data points from 10^−6^ to 1 s) at room temperature. One week after incubation, fully expanded leaflets, were held in darkness for 30 min using clips, and then exposed to a strong red-light pulse (3500 µmol photons.m^−^² s^−^¹) for 1 sec, which provided sufficient photoenergy to saturate all PSII reaction centers. To provide homogeneous illumination, fluorescence-inducing light was supplied by three light-emitting diodes focused on the leaf surface. High-performance PIN photodiode detectors recorded the increase in fluorescence during illumination of the dark-adapted samples. The recorded fluorescence signal was digitized in the control unit using a fast analog-to-digital converter. All data were analyzed using PEA Plus V1.01 software. The equations and definitions of the so-called JIP-test photosynthetic indices calculated in this study (Strasser et al., 2000) are shown in **Table 1**.

**Table 1:** Photosynthetic parameters.

Parameters	Definition
Extracted and technical fluorescence parameters	
Fo	Fluorescence intensity at 50 μs (O step)
FJ	Fluorescence intensity at 2 ms (J step)
FI	Fluorescence intensity at 30 ms (I step)
Fm	Maximal fluorescence intensity (P step)
Fv = Fm–Fo	Maximal variable fluorescence
Fv/Fo = Oxygen Evolving Complex (OEC) activity	Activity of the water-splitting complex on the donor side of the PSII
Fv/Fm = ϕPo	Maximal quantum yield of primary photochemical events
Quantum efficiencies or flux ratios or yields	
ΨEo = ETo/TRo = 1- VJ	Excitation transfer efficiency to the electron transport chain beyond QA
ϕRo = (1 − VJ) · (Fv/Fo)	Quantum yield for the reduction of end acceptors of PSI per photon absorbed
ΦEo = ETo/ABS = 1- (F0/FM) ·ΨEo	Quantum yield of electron transport
ϕDo = Fo/Fm	Quantum yield of energy dissipation (at t=0)
Performance indices	
PI_ABS_ = (RC/ABS)·[ΦPo (1- ΦPo)]· [ψEo/(1- ψEo)]	Performance index

According to Mehta et al., 2010; Oukarroum et al., 2007.

ETo, flux of electron from QA^-^ into the electron transport chain;

ABS, absorption energy flux;

RC, reaction center of PSII;

TRo, excitation energy flux trapped by a RC and utilized for the reduction of QA to QA^-^

### Extraction and quality assessment of total RNA

A total RNA Extraction Kit (Parstous, Iran) was used for RNA extraction. Seventy-two hours after treatment, 40 mg of root tissue was gently ground in liquid nitrogen using a chilled mortar and pestle to obtain a smooth powder. To the powder, 750 µL of lysis buffer was added to disrupt cell membranes and release RNA. The lysis buffer contained guanidine isothiocyanate, sodium dodecyl sarcosine, 2-mercaptoethanol, and sodium citrate. Following the incubation of cell lysate at room temperature for 5 min, 150 µL of chloroform was added to the mixture. The homogenate was vigorously shaken for 15 sec and kept at room temperature for 3 min, followed by centrifugation at 20400 g at 4°C for 12 min (5417R, Eppendorf, Germany). The upper phase was separated and mixed with an equal volume of absolute ethanol. The mixture was transferred to a spin column and centrifuged at 20400 g for 1 min. To ensure that all contaminants such as salts and phenol were removed, 700 µL wash buffer solution containing ethanol and nuclease-free water was used. The purified solution was centrifuged at 20400 g for 1 min., and the residual wash buffer solution was expelled by repeating centrifugation in a new tube at 20400 g for 2 min. Spin columns were then transferred to new microtubes and 30 µL of RNase-free DEPC-treated water was added followed by incubation at room temperature for 3 min to dissolve RNA. RNA was eluted from the columns by centrifugation at 20400 g for 1 min. RNA solutions were stored at -70°C until further analysis.

RNA quality and quantity were assessed by nanodrop spectrophotometry (Lite Plus Microvolume Spectrophotometer, ThermoFisher Scientific, USA) at specific wavelengths, including 230, 260, and 280 nm. The A_260_/A_280_ and A_260_/A_230_ ratios were calculated and the RNA concentration was determined. To visualize RNA integrity, 2% agarose gel electrophoresis was carried out and a Gel Documentation System was used for band intensity assessment (Bio Rad Gel Doc XR System, Universal Hood II, USA).

### cDNA synthesis, primer design, and quantitative real-time reverse transcription PCR

The DNase I kit (ThermoFisher Scientific, USA) was utilized to eliminate genomic DNA contamination in extracted RNAs. An Easy™ cDNA Synthesis Kit (Parstous, Iran) was used to synthesize complementary DNA (cDNA) from total RNA following the manufacturer’s instructions. The subsequent thermal protocol was executed using a thermal cycler (Eppendorf, Mastercycler gradient, Germany): incubation at 25°C for 10 min, followed by 47°C for 60 min, cessation of the reaction by heating to 85°C for 5 min, and subsequent cooling on ice (at 4°C).

cDNA samples were stored at -20°C and optimal annealing temperatures for all primer pairs were determined using a gradient PCR (55-65°C) on the Mastercycler Gradient (Eppendorf, Germany). PCR products were analyzed by 1% agarose gel electrophoresis and UV visualization. Based on the observed bands of amplification products, the annealing temperature of 60°C was chosen for all gene primer pairs (data not shown). Subsequently, cDNA samples were utilized for qRT-PCR using the SYBR Green Real-Time PCR Master Mix (Parstous, Iran). PCR primers for salt-inducible genes *SOS3*, *SOS2*, *SOS1*, *NHX4*, and *NHX2*, as well as for the reference gene *EF1-α* (Elongation factor 1-α) were developed using Beacon Primer Designer 8.1 (Premier Biosoft International, Palo Alto, CA, USA) and Oligo-7 (Molecular Biology Insights, Inc., Cascade, CO, USA). The qRT-PCR reaction mixture contained 10 µL of 2X Master Mix, 1 µL of diluted cDNA at 1:20 ratio (optimized after testing several cDNA stock dilutions of 1:10, 1:20, 1:50, and 1:100 (v/v)). Variable quantities of primer pairs (*SOS3*, *SOS2*, *SOS1*, *NHX4*, *NHX2*, and *EF1-α*) were utilized according to the subsequent configuration for primer volumes (0, 0.5, 1, 2 µL of forward primers paired with 0, 0.5, 1, 2 µL of reverse primers), and PCR-grade distilled water to achieve a final volume of 20 µL. Amplification was conducted utilizing a real-time PCR system (Rotor-Gene Q, Orange Scientific, Belgium) programmed as follows: an initial hold at 94°C for 10 min; a subsequent phase consisting of 35 cycles of denaturation at 95°C for 15 sec, annealing at 60°C for 30 sec, and extension at 72°C for 45 sec; and a final hold at 72°C for 5 min. Melting curves were established between 55 and 95°C. The relative expression levels of genes were quantified in triplicate using the 2^-△△Ct^ method. The *EF1-α* gene was utilized as an internal control for normalizing sample variations (Livak and Schmittgen, 2001).

### Statistical analysis

All measurements were conducted in four replicates. Shapiro-Wilk and Levene’s tests were utilized to evaluate the normality and homogeneity of variances. Differences among group means were evaluated using the analysis of variances (ANOVA) with R software, version 2025.09.1 + 401. To identify specific treatments with significant variations, the Tukey *post-hoc* test was applied at a significance level of p ≤ 0.05.

## Results

### Characterization of CDs

**Figure 1:**
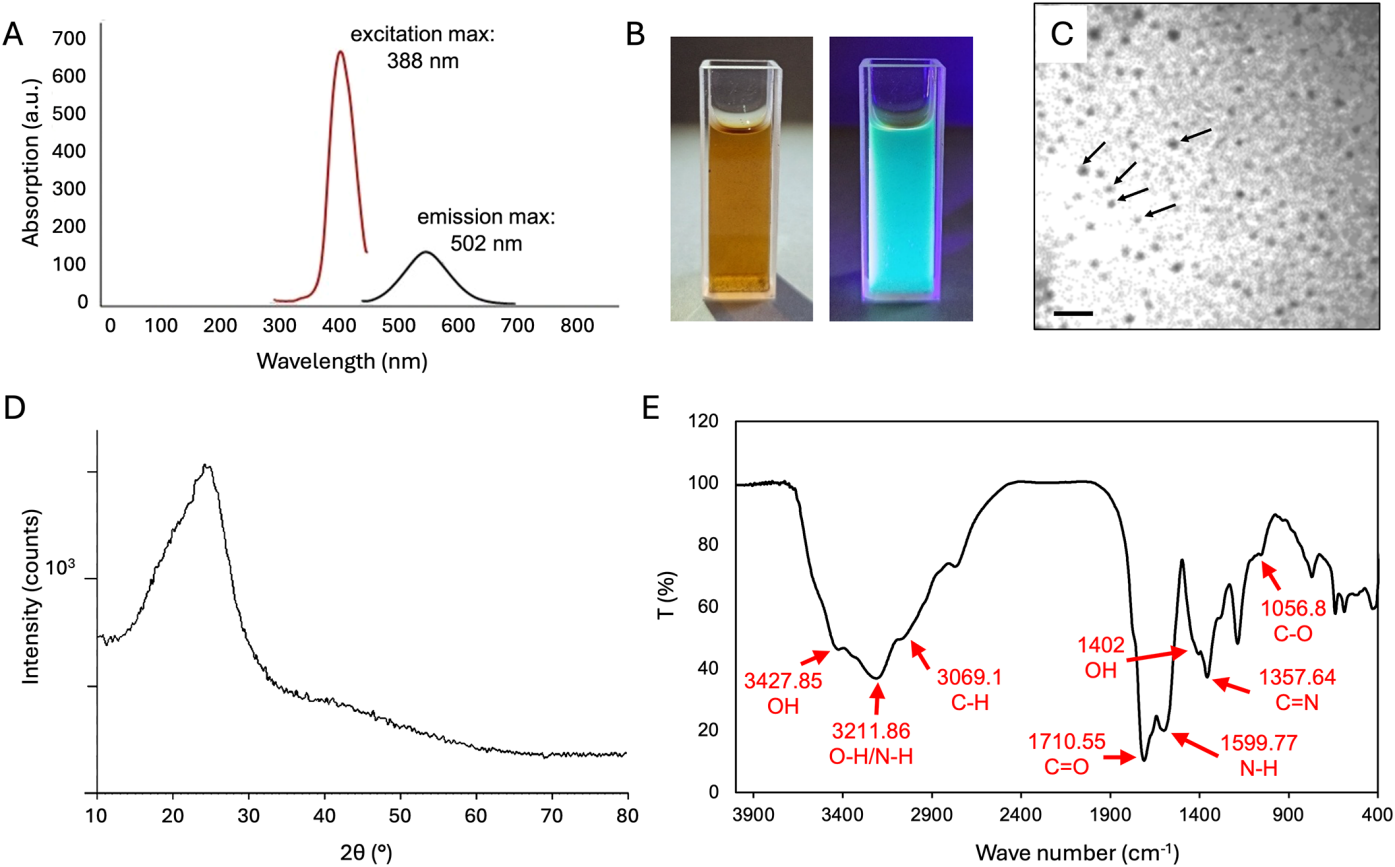
Characteristics of CDs produced by the hydrothermal method. **(A)** Absorption spectrum from 300 to 700 nm (red curve) and fluorescence emission spectrum from 400 to 650 nm (black curve) of CDs in aqueous solution; **(B)** Macroscopic image of hydrothermally synthesized carbon dots in aqueous solution under visible light (left) and ultraviolet radiation (right); **(C)** Transmission Electron Micrograph of CD particles. Black arrows indicate individual carbon dot particles; **(D)** X-ray diffraction (XRD) spectrum; **(E)** Spectrum of Fourier Transform Infrared (FTIR) analysis. Size bar in **(C)**, 25 nm.

Hydrothermally synthesized carbon dots (CDs) exhibited an absorption peak at 388 nm, and an emission maximum at 502 nm (**Figure 1A**), resulting in a strong green-blue fluorescence (**Figure 1B**). TEM imaging revealed that CD particles exhibited a roughly spherical shape with a diameter less than 10 nm (**Figure 1C**). X-ray diffraction (XRD) analysis showed that the CDs had a broad peak at 2θ ~ 25° (**Figure 1D**). The surface charge of CDs was measured as a criterion for inter-particle electrostatic repulsion. The results showed a zeta potential of around -0.2 mV. The FTIR spectrum of CDs showed different peaks at various wave numbers (**Figure 1E**), each corresponding to specific functional groups on the surface of CDs: The peaks (and the corresponding functional groups) were at 3427.85 (-OH), 3211.86 (-OH or N-H), 3069.16 (C-H), 1710.55 (C=O), 1599.66 (N-H), 1402 (-OH), 1357.64 (C=N) and 1056.8 cm^−^¹ (C-O).

### CD uptake and transport in tomato seedlings

**Figure 2:**
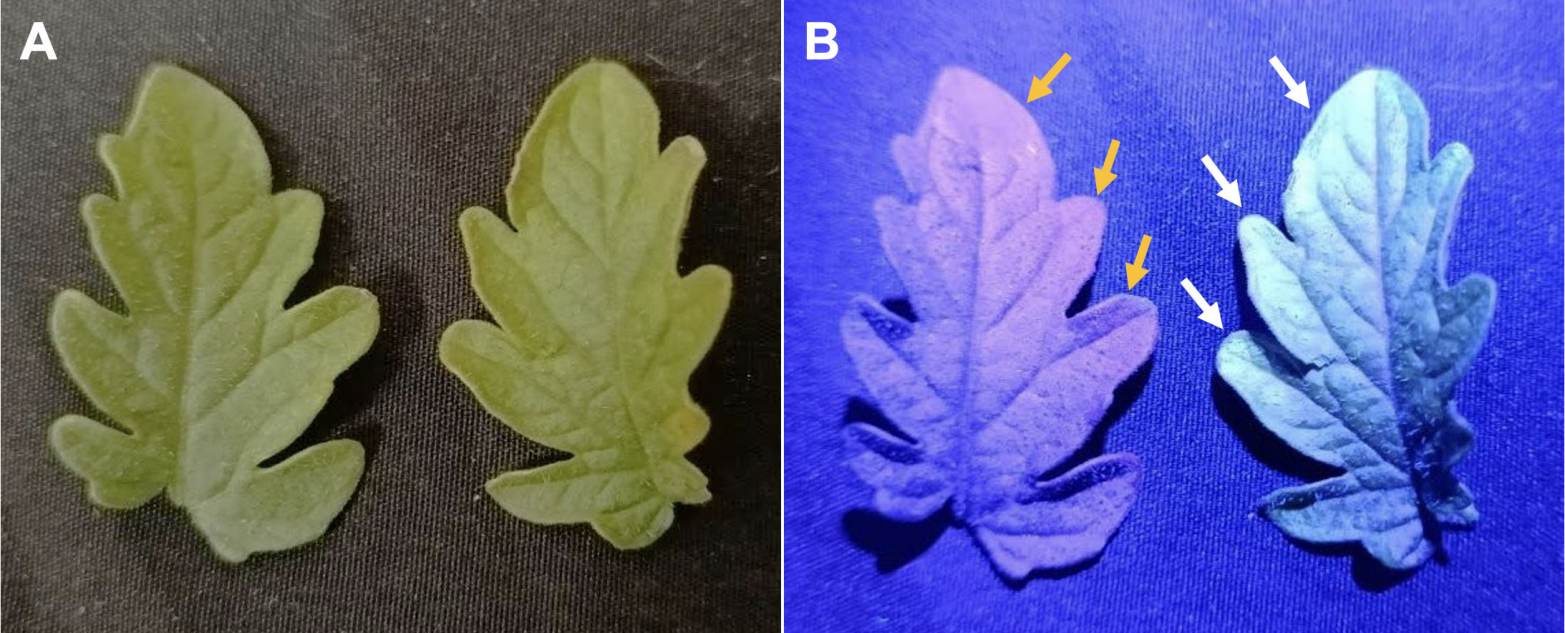
Accumulation of hydroponically applied CDs in tomato leaves. Plants were treated from the root system with 30 mg·L^-1^ CDs for one week. Detached leaves (control: left; CD-treated: right) were imaged under visible light **(A)** and UV light **(B)**.

To be physiologically effective, CDs must be taken up, i.e. they must pass the cell walls of root cells and be transported within the plant. To test the potential of CDs to be taken up and transported, we examined their presence in leaves after hydroponic administration from the root system. Taking advantage of their strong fluorescence (**Figures 1A, B**; Abbas et al., 2025), we imaged a leaflet each from a CD-treated plant and a control plant using visible illumination (**Figure 2A**) and UV excitation (**Figure 2B**). Strong blue-green fluorescence revealed accumulation of CDs with highest levels at the leaf margin (**Figures 2B**, right), while the control showed only the purplish chlorophyll fluorescence typical of green tissues (**Figures 2B**, left). These results show that CDs can penetrate roots, enter the transpiration stream and accumulate in leaves.

### Evaluation of appropriate CD and NaCl concentrations

**Figure 3:**
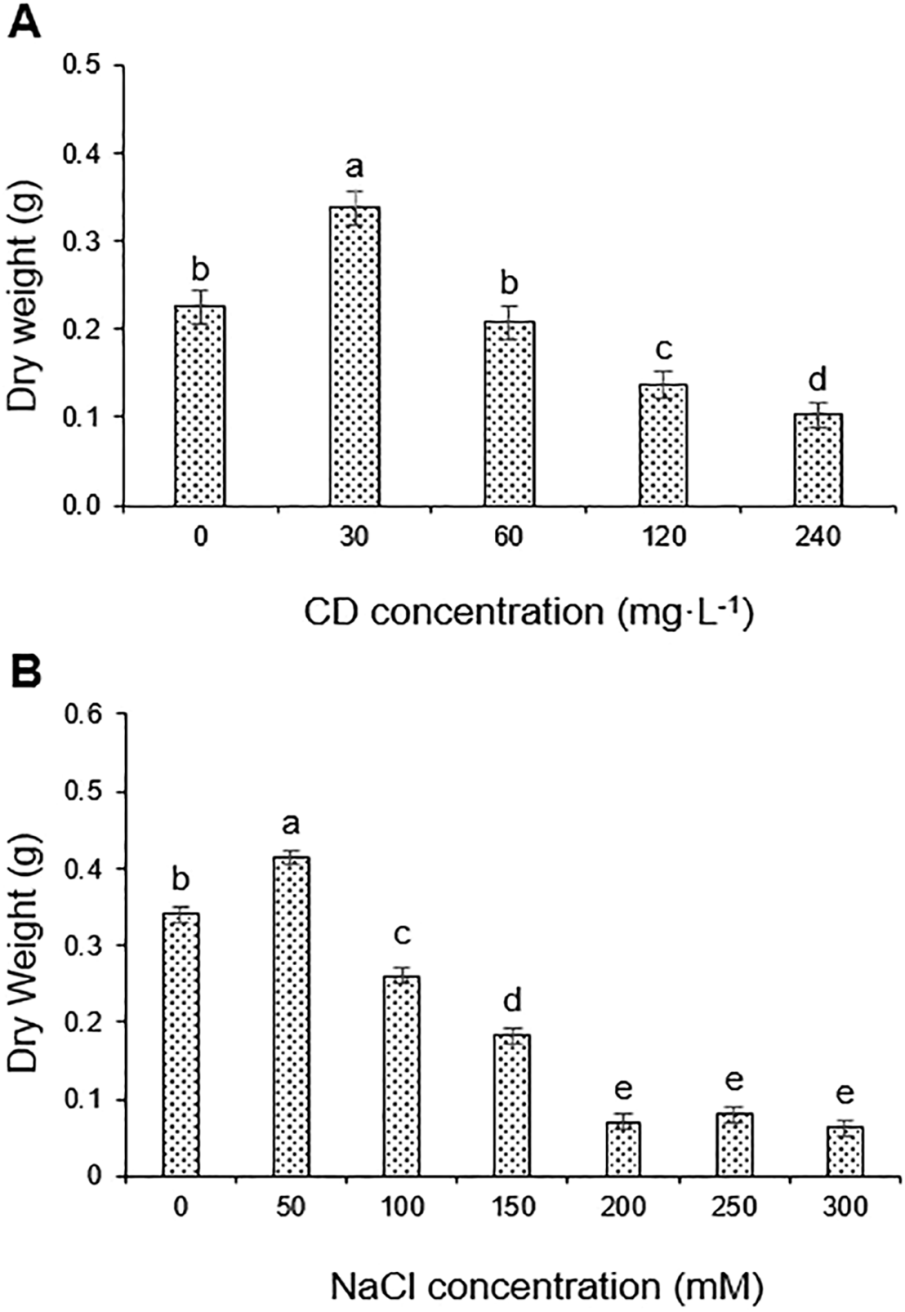
Effect of NaCl and CDs on growth of tomato plants. **(A)** Dry weight of plants treated with NaCl at concentrations of 0, 50, 100, 150, 200, and 300 mM; **(B)** Dry weight of plants treated with CDs at concentrations of 0, 30, 60, 120, and 240 mg·L^-1^. Data are presented as the mean ± standard error (n=4). Different letters indicate statistically significant differences as determined by one-way ANOVA (p ≤ 0.05).

To determine appropriate conditions for treatments of tomato plants with CDs and NaCl, we performed two experiments with concentration series. Plants were separately treated with up to 240 mg·L^−^¹ CDs and with 0–300 mM NaCl (**Figure 3**). Plants treated with 30 mg·L^−^¹ CDs showed significant growth promotion, whereas levels higher than 120 mg·L^−^¹ were inhibitory (**Figure 3A**). Salt treatments had negative effects at concentrations of 100 mM and more (**Figure 3B**). Based on these results, we considered the limited stress effects caused by 100 and 150 mM NaCl as suitable and realistic conditions to test potential beneficial effects of CDs applied at a concentration of 30 mg·L^−^¹.

### CDs protect plants against salt stress

**Figure 4:**
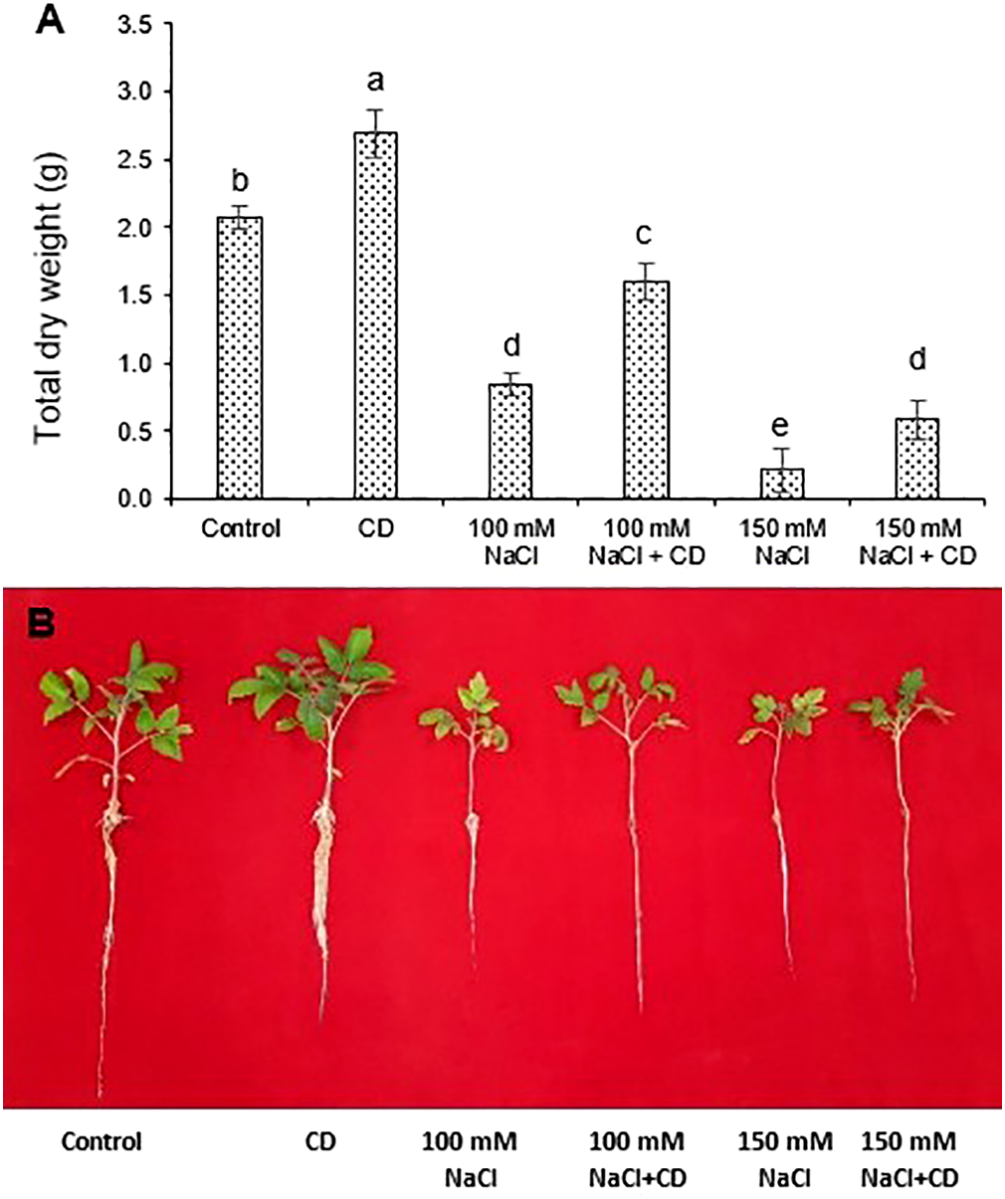
Effect of CDs and salinity stress on growth of tomato. **(A)** Dry weight of tomato plants hydroponically cultured with CDs (30 mg·L^-1^), salinity stress (100 and 150 mM NaCl), or with their combination. Data are presented as the mean ± standard error (n=4). Different letters indicate statistically significant differences as determined by one-way ANOVA (p ≤ 0.05). **(B)** Appearance of tomato plants treated as in **(A)**.

Combined treatments of plants with 100 mM NaCl and 30 mg·L^-1^ CDs, increased plant growth by 84% relative to salt alone (**Figures 4A, B**), indicative of significant protection against salt stress. The severe growth retardation caused by 150 mM NaCl treatment was also mitigated by CDs but to a lesser extent than in the case of 100 mM NaCl (**Figures 4A, B**). Thus, moderate salt stress can be alleviated by CDs.

**Figure 5:**
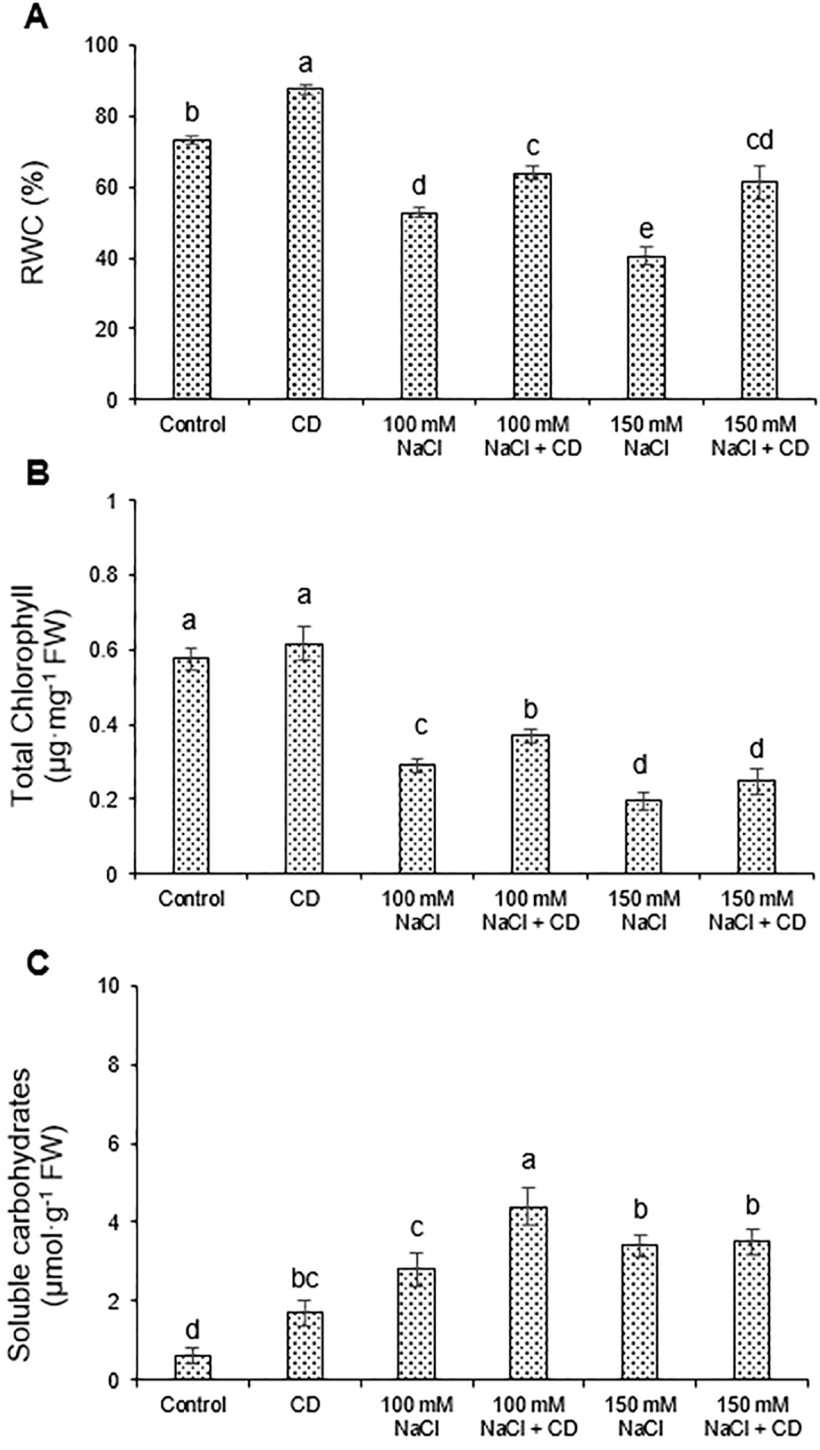
Effect of CDs and salinity stress on water, chlorophyll, and carbohydrate content in leaves. **(A)** Relative water content (% RWC), **(B)** total chlorophyll content, and **(C)** soluble carbohydrate content in leaves of tomato plants hydroponically cultured with CDs (30 mg·L^-1^), salinity stress (100 and 150 mM NaCl), or with their combination. Data are presented as the mean ± standard error (n=4). Different letters indicate statistically significant differences as determined by one-way ANOVA (p ≤ 0.05).

To explain the growth effects caused by salt and CDs, we determined physiologically relevant parameters such as relative water content (RWC), chlorophyll and carbohydrate content, which are known to be affected by salinity (Boussora et al., 2024; Zhang et al., 2024). Salt stress considerably decreased RWC at 100 and 150 mM, an effect that was reversed by CDs by approximately 20% and 50%, respectively, relative to plants subjected to 100 and 150 mM NaCl alone (**Figure 5A**). Negative effects of salt on chlorophyll content were mitigated by CDs, although the protective effect at the higher salt level was not statistically significant (**Figure 5B**). Soluble carbohydrates are central osmoprotectants (Liu et al., 2022), and were therefore assessed here. Their levels were increased both, by CDs and by salt alone, and in an additive fashion in the combined treatment (100 mM NaCl+CDs). This suggests a protective effect of CDs by increasing the accumulation of osmoprotective solutes, an effect that was not observed, however, at the high salt level (150 mM NaCl) (**Figure 5C**).

**Figure 6:**
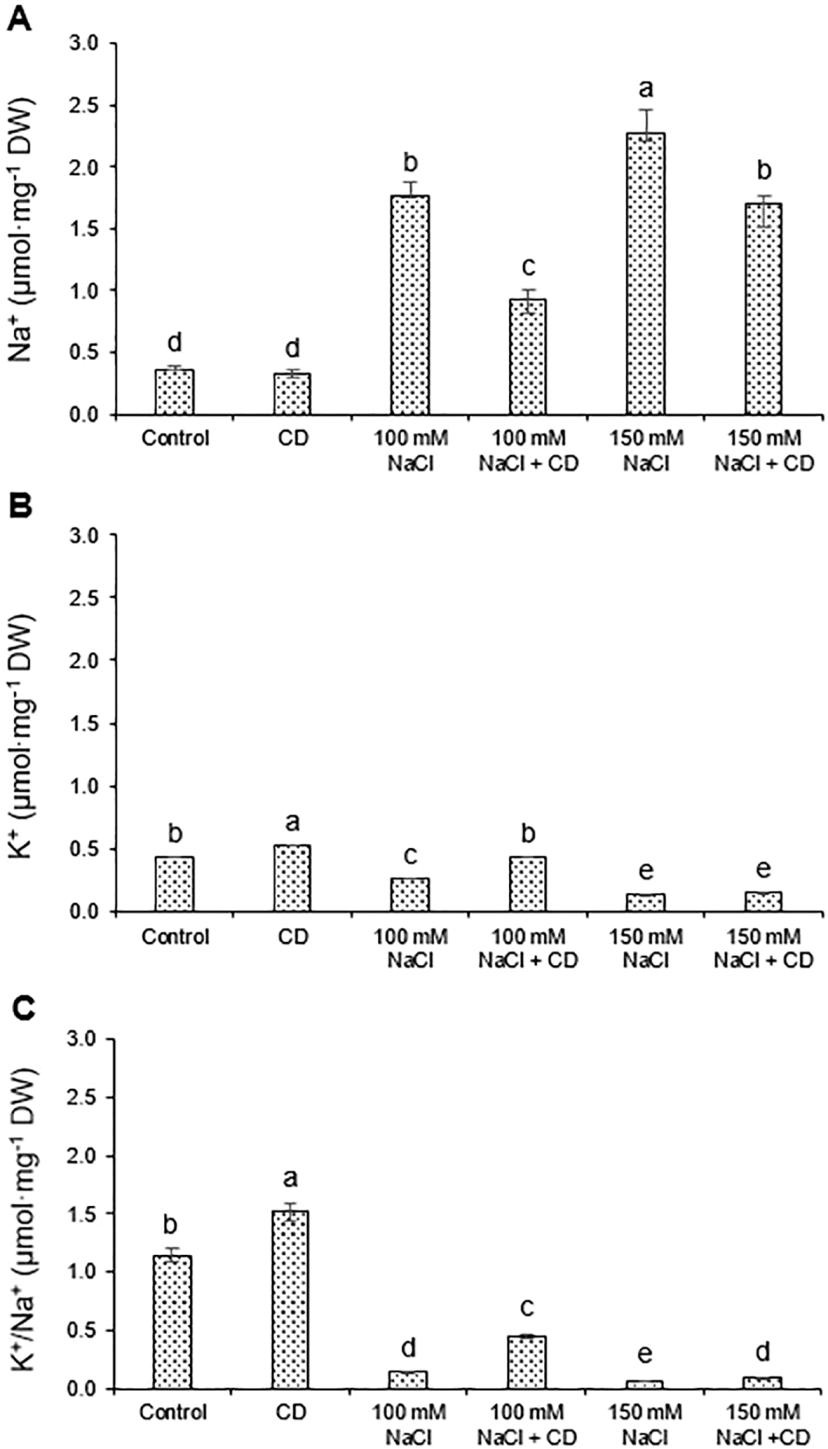
Effect of CDs and salinity stress on Na^+^ and K^+^ content in tomato roots. Content in Na^+^ **(A)** K^+^ **(B)** and K^+^/Na^+^ ratio **(C)** in roots of tomato plants hydroponically cultured with CDs (30 mg·L^-1^), salinity stress (100 and 150 mM NaCl), or with their combination. Data are presented as the mean ± standard error (n=4). Different letters indicate statistically significant differences as determined by one-way ANOVA (p ≤ 0.05).

As a measure of ion balance, we determined the tissue content of Na^+^ and K^+^ in the roots. Salt stresses of 100 and 150 mM NaCl caused a considerable increase in Na^+^ levels (**Figure 6A**) and a concomitant decrease in K^+^ content (**Figure 6B**). The simultaneous application of CDs and NaCl significantly reduced the Na^+^ content by almost 50% relative to salt alone (**Figure 6A**) and increased K^+^ accumulation (**Figure 6B**) by around 75% compared to 100 mM salt. The resulting K^+^/Na^+^ ratios strongly decreased in plants subjected to salt stress (**Figure 6C**), an effect that was counteracted by CDs at 100 mM NaCl (**Figure 6C**).

### CDs have beneficial effects on photosynthetic parameters

Fluorescence of chlorophyll *a* is a reliable parameter to characterize the status of the photosynthetic electron transport chain (Strasser et al., 2000; Swoczyna et al., 2022). Parameters calculated based on fluorescence of chlorophyll *a* in the PSII reaction center included Fv/Fo, Fv/Fm, ΨEo, ϕEo, ϕRo, ϕDo, and PI_ABS_ (**Table 1**). Fv/Fo is a criterion for evaluating the performance of the PSII water-splitting complex also known as the oxygen-evolving complex (OEC). Both levels of salinity stress reduced Fv/Fo, an effect that was reversed by CD treatment by nearly 100% relative to the salt stress conditions (**Table 2**). Similarly, both salt stress levels reduced the maximal quantum yield of primary photochemical events (Fv/Fm), excitation transfer efficiency to the electron transport chain (ΨEo), electron transport quantum yield (ϕEo), and quantum yield of the reduction of end electron acceptors at the PSI acceptor site (ϕRo) (**Table 2**). In all these cases, concomitant application of 30 mg·L^-1^ CDs significantly alleviated the stress symptoms and improved photosynthetic parameters to varying degrees (**Table 2**). Conversely, the quantum yield of energy dissipation (ϕDo) was significantly increased by salt stress, and this stress symptom was reversed by CD application (**Table 2**). Finally, PI_ABS_, which represents the performance index of PSII, significantly decreased under salinity stress, an effect that was reversed by CD application (**Table 2**).

**Table 2:** Effect of salt and CDs on photosynthetic parameters.

Fluorescence parameters	Treatments					
	Control	CD	100 mM NaCl	100 mM NaCl + CD	150 mM NaCl	150 mM NaCl + CD
Fv/Fo (OEC)	6.92 ± 0.25^b^	9.87 ± 0.39^a^	3.79 ± 0.07^e^	6.19 ± 0.15^c^	2.16 ± 0.09^f^	4.27 ± 0.12^d^
Fv/Fm	0.87 ± 0.0038^b^	0.91 ± 0.0033^a^	0.79 ± 0.0032^c^	0.86 ± 0.0028^b^	0.68 ± 0.0088^d^	0.81 ± 0.0044^c^
ΨEo	0.5 ± 0.0125^b^	0.6 ± 0.0125^a^	0.3 ± 0.0129^e^	0.42 ± 0.0138^c^	0.25 ± 0.0132^f^	0.36 ± 0.0125^d^
ϕEo	0.43 ± 0.0105^b^	0.54 ± 0.0115^a^	0.24 ± 0.0105^d^	0.36 ± 0.0127^c^	0.17 ± 0.0071^e^	0.293 ± 0.0092^c^
ϕRo	3.44 ± 0.13^b^	5.90 ± 0.26^a^	1.14 ± 0.06^e^	2.62 ± 0.14^c^	0.53 ± 0.01^f^	1.55 ± 0.04^d^
φDo	0.13 ± 0.0038^d^	0.09 ± 0.0033^e^	0.21 ± 0.0032^b^	0.14 ± 0.0028^d^	0.32 ± 0.0088^a^	0.19 ± 0.0044^c^
PI_ABS_	6.56 ± 0.4489^b^	9.91 ± 0.7012^a^	1.3 ± 0.0819^d^	3.89 ± 0.3378^c^	0.32 ± 0.0274^e^	1.31 ± 0.0441^d^

Fluorescence parameters were extracted from OJIP chlorophyll *a* transient analysis of tomato under various treatments including hydroponically applied CDs (30 mg·L^-1^), salinity stress (100 and 150 mM NaCl), and the combined treatment of CDs and salinity. Data are presented in arbitrary units as the mean ± standard error (n=4). Different letters indicate statistically significant differences as determined by one-way ANOVA (p ≤ 0.05).

Strikingly, application of 30 mg·L^-1^ CDs alone improved most photosynthetic parameters relative to controls (**Table 2**). In particular, Fv/Fo (OEC), Fv/Fm, ΨEo, ϕRo, and PI_ABS_, were increased by CDs, whereas ϕDo decreased in CD-treated plants compared to controls (**Table 2**). These effects may contribute to the growth stimulation mediated by CDs at low levels (**Figure 3A**).

### CDs attenuate expression of salt-responsive genes

**Figure 7:**
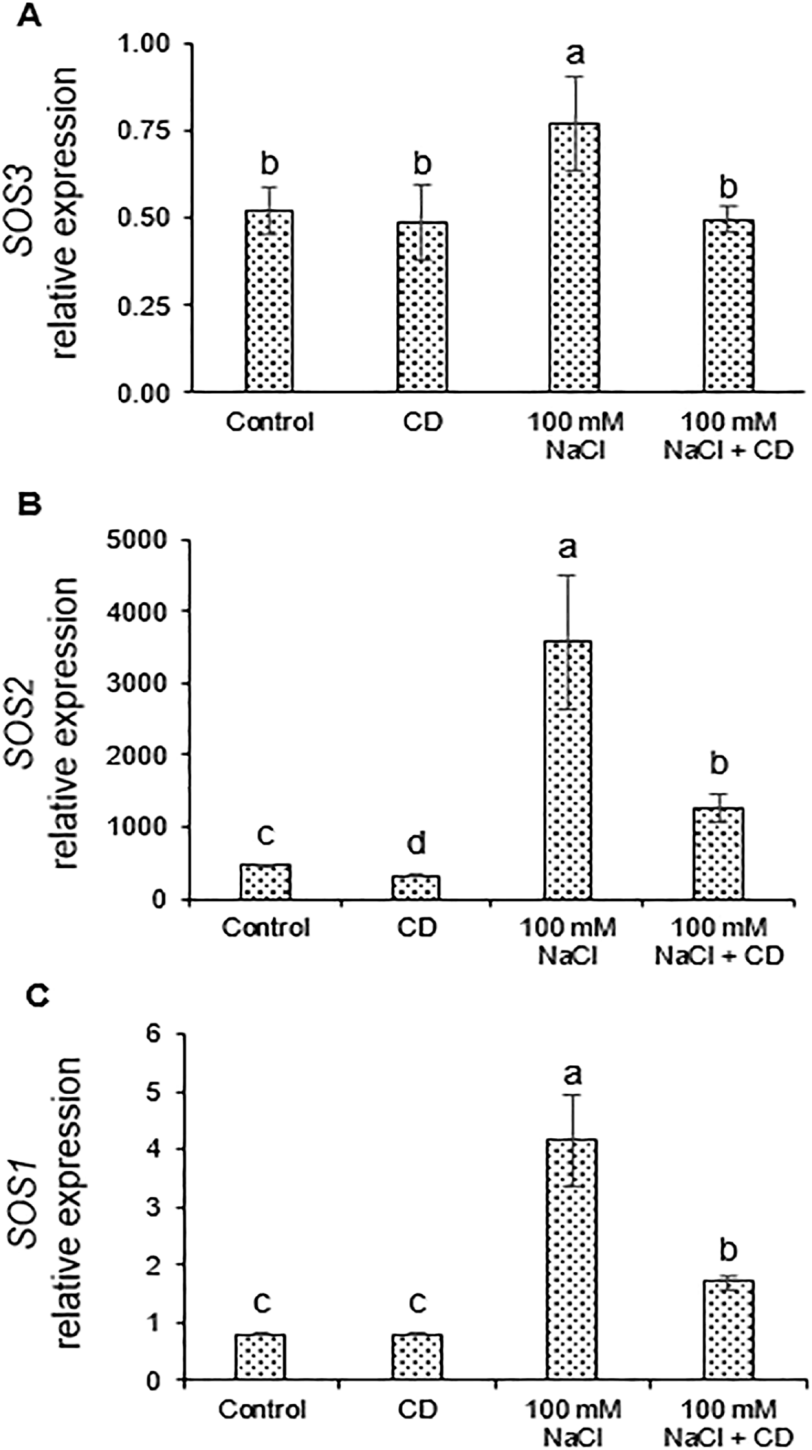
Effect of CDs and salinity stress on expression of *SOS* genes in tomato roots. Expression of *SOS3* **(A)**, *SOS2* **(B)**, and *SOS1* **(C)** normalized to the *EF1-α* reference gene in roots of tomato plants hydroponically cultured with CDs (30 mg·L^-1^), salinity stress (100 and 150 mM NaCl), or with their combination. Data are presented as the mean ± standard error (n=4). Different letters indicate statistically significant differences as determined by one-way ANOVA (p ≤ 0.05).

**Figure 8:**
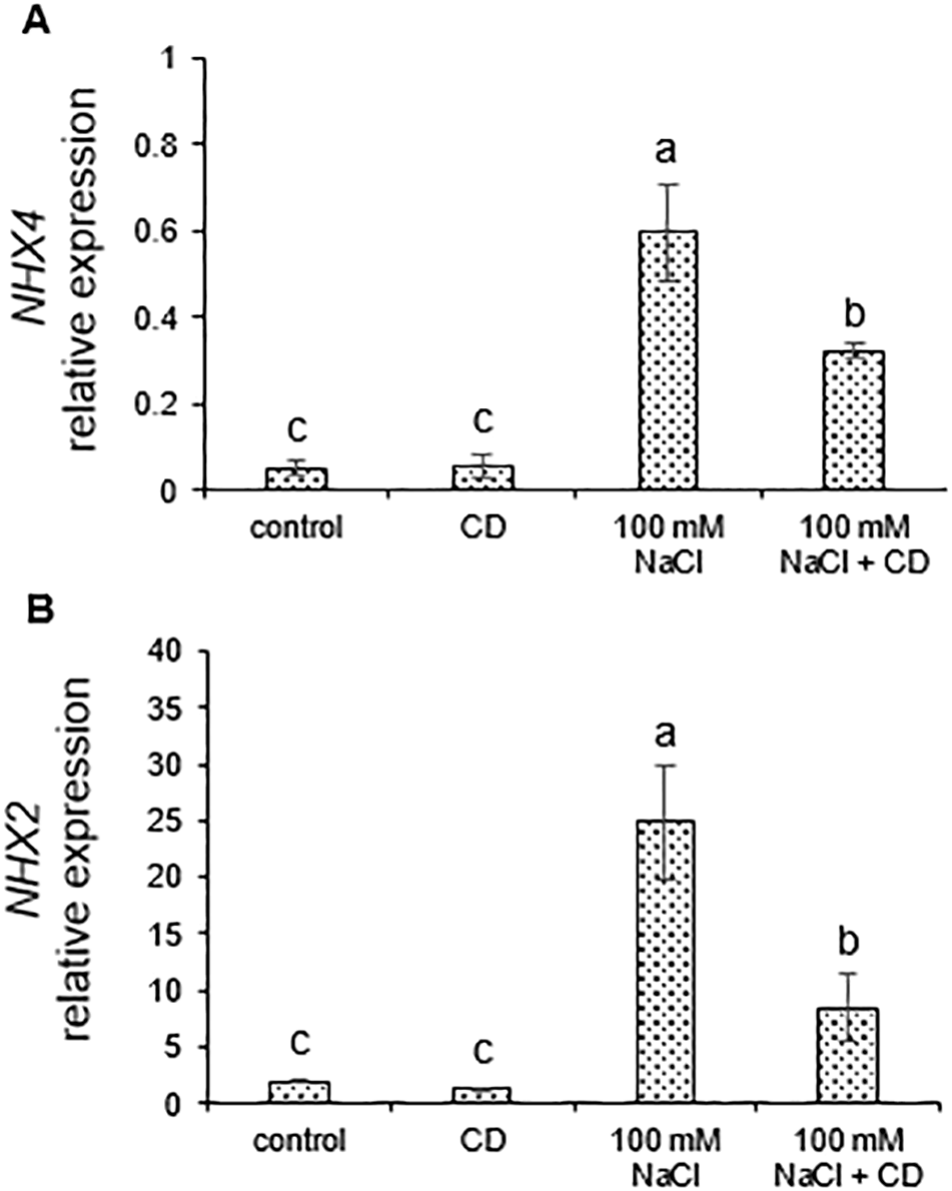
Effect of CDs and salinity stress on expression of *NHX* genes in tomato roots. Expression of *NHX4* **(A)** and *NHX2* **(B)** normalized to the *EF1-α* reference gene in roots of tomato plants hydroponically cultured with CDs (30 mg·L^-1^), salinity stress (100 and 150 mM NaCl), or with their combination. Data are presented as the mean ± standard error (n=4). Different letters indicate statistically significant differences as determined by one-way ANOVA (p ≤ 0.05).

*SOS* genes are among the central salt-responsive genes of plants (Ali et al., 2023). We quantified the expression of *SOS1*, *SOS2*, and *SOS3*, as well as of the ion transporters *NHX2* and *NHX4*, by quantitative real-time RT-PCR (qPCR) using the primers listed in **Table 3**. *SOS1*, *SOS2*, and *SOS3* were coordinately induced by salt, while CDs did not induce *SOS* genes on their own (**Figures 7A–C**). The combined treatment with salt and CDs resulted in lower expression than in the case of salt alone (**Figures 7A–C**). Similarly, *NHX2* and *NHX4* were strongly induced by salt, but unaffected by CDs alone (**Figures 8A, B**). In the combined treatment with salt and CDs, *NHX2* and *NHX4* were expressed at lower levels than in plants treated with salt alone (**Figures 8A, B**).

**Table 3:** List of primers used for qPCR.

Genes	Gene ID	Primers sequences (5′→3′)	PCR product (bp)
*SOS3*	NM_001247776.3	F: TTTACGGTGAGTGAAGTTGAAGCATTA R: TTCTTCCTTGTGAATTAGTCCATCATCAAT	90
*SOS2*	NM_001247281.2	F: GATTTATTTCCCGCCAACCTGCTA R: CCAGCCCTATTTGCCGTTACC	130
*SOS1*	NM_001247769.3	F: TGGCTATGGTTTGGACTTGAAAGA R: CACTGGAACGCTTGACTGACA	101
*NHX4*	NM_001246956.2	F: AATCCCAGTATAGCCCTCAGTTTCC R: AATGTAAGCACTAAGAAGACCAGTTCCT	99
*NHX2*	NM_001328634.1	F: GAACCGTCATCAGGGAACAGG R: TTGGCTCATCTTCATCTTCGTCTC	139
*EF1-α*	NM_001247106.2	F: TGCTGCTGTAACAAGATGGATGCTA R: CTCCTTCAAAACCAGAGATTGGAACAAAG	148

Taken together, these results show that CDs reduce the induction of salt-responsive genes. This is consistent with a protective role of CDs which reduces salt stress, thereby attenuating induction of salt-responsive genes.

## Discussion

### Physico-chemical characteristics of CDs

We have synthesized carbon dots (CDs) in a size range of 5–10 nm, and with a regular roundish shape, although the wide peak appearing at 2θ ~25° in their XRD spectrum suggests a random and amorphous structure (Moseenkov et al., 2023). Reactive chemical groups identified by FTIR included hydroxyl (-OH), amino (N-H), carbonyl (C=O), C-H, C=N and C-O bonds as main functional groups on the CD surface (**Figure 1E**) (Pavia et al., 2001; Nandiyanto et al., 2023). The -CO-NH_2_ functional groups may be responsible for the strong green fluorescence (**Figure 1**) (Ding et al., 2018), and the carboxyl and hydroxyl groups are likely to contribute to the negative zeta potential (-0.2 mV), which creates a repulsive force among nanoparticles, thus increasing their stability and counteracting aggregation (Tantra et al., 2010; Sharma, 2019). The quantum dimensions of CD particles of <10 nm (Maholiya et al., 2023), may contribute to their efficient uptake and transport from the roots to the leaves (**Figure 2**) (Choi et al., 2007).

### CDs can protect plants from salt stress

Salinity interferes with numerous biochemical and physiological processes (Hao et al., 2021) and limits plant growth by disrupting osmotic balance (Singh et al., 2022). Increased salinity elevates Na^+^ concentrations in the plant, while reducing K^+^ levels, ultimately inhibiting plant growth due to ion imbalance (Munns and Tester, 2008; Hao et al., 2021). In addition, salinity lowers the water potential around the roots and triggers stomatal closure (Balasubramaniam et al., 2023). Finally, growth under salt stress is attenuated by a decrease in tissue water content and photosynthetic activity. All these symptoms of salt stress were observed in tomato seedlings subjected to 100 mM or 150 mM NaCl (**Figures 3**-**6**; **Table 2**).

CD treatment alleviated most assessed salt stress parameters and reversed the negative growth effect caused by salt (**Figures 3**-**6**; **Table 2**), suggesting that CDs represent an appropriate remedy to protect crops against salt stress, although the exact mode of action remains to be elucidated. CDs can potentially act by inducing aquaporin genes and improve water uptake (Kou et al., 2021). Considering the positive effects of CDs on chlorophyll, CDs may upregulate genes encoding chlorophyll synthase (Li et al., 2021), resulting in enhanced chlorophyll biosynthesis (Hualpa-Ramirez et al., 2024; Borghesi et al., 2011).

Salt stress can also be reduced by osmolytes such as carbohydrates, low-molecular-weight neutral solutes that promote water retention in saline environments with highly negative osmotic potential (Liu et al., 2022). Indeed, our results showed an increase in soluble carbohydrates in response to NaCl stress. Under extreme conditions, increased investment in osmolytes may contribute to reduced growth in salt-stressed plants, due to diversion of resources from growth to survival mechanisms (Thalmann and Santelia, 2017; Atta et al., 2023; Liang et al., 2023). In our experiments, CD treatment enhanced carbohydrate levels both in controls and in salt stressed plants.

As a central parameter of salt stress, Na^+^ levels are increased and K^+^ levels decreased (**Figure 6**). Na^+^ inhibits numerous key enzymes in plant cells (Debouba et al., 2006; Youssef et al., 2022). On the other hand, K^+^ is one of the central compatible solutes involved in osmotic regulation and in various physiological processes (Wang et al., 2013; Cui and Guillaume, 2021). Therefore, the K^+^/Na^+^ ratio in plant tissues is a critical parameter and indicator of salt stress resistance (Isayenkov, 2012; Chakraborty et al., 2020). We found that CDs increased the K^+^/Na^+^ ratio in the root system (**Figure 6**). Recent evidence showed that CDs can upregulate potassium uptake-related genes such as inward rectifying K^+^ channel NKT1 (Chen et al., 2020), and K^+^ transporter NtHAK1 (Chen et al., 2020; Zhao et al., 2024), which may contribute to restored K^+^/Na^+^ homeostasis in our experiment, thereby supporting water uptake and plant cell turgor (Mohammadi et al., 2024; Rizk et al., 2024).

### CDs have beneficial effects on photosynthetic machinery

Chlorophyll *a* fluorescence is a quick, precise, non-invasive, and reliable readout for the status of the photosynthetic apparatus in response to environmental conditions (Strasser et al., 2000). Salt stress can alter the structure and performance of the photosynthetic apparatus (Mane et al., 2010) leading to a decline in PSII performance and photosynthetic efficiency (Yan et al., 2013). Conversely, CDs have been shown to positively influence photosynthetic performance metrics (Li et al., 2020b) as well as membrane stability (Ji et al., 2022). We show here that CD treatment increases OEC activity (Fv/Fo) under salt stress and normal conditions (**Table 2**).

Salt stress also led to a decrease in the maximal quantum yield of primary photochemical events (Fv/Fm) (**Table 2**), indicating that antenna chlorophylls may lose part of their capacity to fully transfer absorbed photon energy to reaction centers, thereby reducing the energy trapped by antenna chlorophylls in PSII (Baker, 2008). The application of CDs under salt stress improved Fv/Fm compared to salt stress alone (**Table 2**), presumably by enhancing light conversion and utilization (Li et al., 2020b).

We observed that ΨEo (excitation transfer efficiency to the electron transport chain beyond the PSII electron acceptor plastoquinone QA), ϕEo (quantum yield of electron transport), and ϕRo (quantum yield of the reduction of end electron acceptors at the PSI acceptor site) declined under salinity stress (**Table 2**). The reduction in ΨEo may be attributable to fewer available electron acceptors within the photosynthetic apparatus and a disrupted electron transfer chain (Strasser et al., 2004; Mehta et al., 2010). A decline in ϕEo suggests impaired electron flow beyond plastoquinone QB, a PSII electron carrier after the first electron acceptors, pheophytin (PheoD1) (Klimov, 2003) and plastoquinone QA (De Causmaecker et al., 2019) and possibly a reduced number of electron carriers under salt stress (Mehta et al., 2010). Furthermore, a decrease in ϕEo could reduce the quantum yield of reducing end electron acceptors at the PSI acceptor side (ϕRo).

The observation that the quantum yield of energy dissipation (ϕDo) was increased under salinity stress, complements the reduction of the above-mentioned parameters, since it documents the loss of energy via a non-productive way by reduced light energy trapping, impaired electron transfer, and reduced photochemical activity (Chen and Cheng, 2010). The performance index (PI_ABS_) combines the number of active reaction centers, energy trapped by reaction centers, and the quantum yield of electron transport in PSII (Schreiber et al., 2012). We observed a decrease in PI_ABS_ of salt-stressed plants, potentially caused by photo-inhibition, as well as structural damage to PSII (Ashraf and Harris, 2013; Murata et al., 2007; Takahashi and Murata, 2008). CDs restored PI_ABS_ values in salt-stressed plants, as well as in controls (**Table 2**).

### CDs reduce expression of salt-responsive genes in stressed plants

Plants have conserved mechanisms to remove sodium cations from the cytoplasm such as the SOS pathway and NHX proteins, which function by mediating Na^+^ compartmentalization and extrusion (Gupta and Huang, 2014; Zhou et al., 2024). We observed induction of SOS pathway-related genes within 72 h of salt exposure in tomato roots, confirming their role in salt-stress response. The upregulation of *SOS1*, *NHX4*, and *NHX2* may contribute to Na^+^ detoxification through ion efflux and intracellular compartmentalization (Cavusoglu et al., 2023; Belver et al., 2012).

When salinity was combined with CDs, the induction of all tested salt-responsive genes (*SOS3*, *SOS2*, *SOS1*, *NHX4*, and *NHX2*) was reduced compared to salt stress alone, suggesting that CDs alleviate salt stress. Consistent with this interpretation, physiological parameters including biomass, chlorophyll content, water status, carbohydrate levels, ion balance, and photosynthetic performance were improved in CD-treated plants under salinity (see above). CDs enhanced water content, partially restored the K^+^/Na^+^ ratio, and promoted the accumulation of compatible solutes, resulting in reduced Na^+^ toxicity and ionic imbalance. We conclude that the reduced induction of salt-responsive genes upon CD application is a consequence of the generally increased tolerance to salt stress and the readjustment of the K^+^/Na^+^ ratio.

Whether the protective mechanism of CDs against salt involves the SOS pathway or NHX proteins is unknown, because their regulation involves posttranslational modifications such as phosphorylation. Hence, despite the reduced expression of all salt responsive genes in the combined treatments, relative to salt alone, the SOS pathway and NHX transporters could contribute to the protective effect mediated by CDs.

## Conclusion

We and others have shown that CDs can protect plants from salt stress. However, CDs also had beneficial effects on their own in the absence of salt stress, suggesting that plants under standard culture conditions have an unexplored growth potential that can be harnessed by application of appropriate CD formulations. Although this phenomenon is reminiscent of the effect of plant-growth-promoting rhizobacteria (PGPRs), the mechanistic basis of CD effects is unknown. We show that several photosynthetic parameters are improved (**Table 2**), while dry weight, relative water content, and soluble sugar levels were also increased (**Figures 4**, **5**). These results indicate that CDs may act at the level of chloroplasts. However, other pleiotropic effects on ion balance and plant physiology cannot be excluded. Future research should address the molecular mechanisms by which CDs act on plants. Furthermore, the potential of CDs as sustainable growth-promoters and stress protectors in crop production should be further explored.

## Data Availability

The original contributions presented in the study are included in the article/supplementary material. Further inquiries can be directed to the corresponding authors.
